# Clinical value of MRI in evaluating and diagnosing of humeral lateral condyle fracture in children

**DOI:** 10.1186/s13018-021-02726-6

**Published:** 2021-10-18

**Authors:** Yang Qi, Lin Guo, Man Sun, Zhi Wang

**Affiliations:** Department of Radiology, Tianjin Hospital, No. 406, Jiefang Nan Road, Hexi District, Tianjin, 300211 China

**Keywords:** HLCFs, MRI, Radiograph, Sensitivity, Clinical value

## Abstract

**Background:**

Humeral lateral condyle fractures (HLCFs) are common paediatric fractures. Radiographs are hard to accurately evaluate and diagnose the damage of articular epiphyseal cartilage in HLCFs.

**Methods:**

60 children who should be suspected to be HLCFs in clinical practice from Dec 2015 to Nov 2017 were continuously included as the first part patients. Subsequently, 35 HLCFs patients with complete follow-up information who had no obvious displacement on radiograph were the second part patients. The sensitivity and specificity of radiograph and MRI in diagnosing of HLCFs and their stability were calculated respectively. Calculated the sensitivity and specificity of each scan sequence of MRI in diagnosing of HLCFs osteochondral fractures. The degree of fracture displacement was measured respectively. Compared the ratio of surgical treatment, secondary fracture displacement and complications between the stable fracture group and the unstable fracture group on MRI in part 2 patients.

**Results:**

Sensitivity of diagnosing HLCFs by MRI was significantly higher than radiograph (100.00% vs. 89.09%, *P* = 0.03). Sensitivity of diagnosing integrity of trochlear cartilage chain by MRI was 96.30%, which was significantly higher than that by radiograph (62.96%, *P* < 0.01). The sensitivity of cartilage sensitive sequence (3D-FS-FSPGR/3D-FSPGR) was different with FS-PDWI and FS-T2WI (*P* = 0.01 and *P* = 0.02, respectively). The degree of HLCFs displacement by MRI was higher than radiograph (*P* < 0.05). In the unstable fracture group, 5 cases (45.45%) had a fracture displacement of more than 2 mm on MRI, which was significantly higher than that in stable fracture group (0.00%, *P* < 0.01).

**Conclusions:**

MRI is superior to the radiograph of elbow joint in evaluating and diagnosing children HLCFs and their stability. The coronal 3D-FS-FSPGR/3D-FSPGR sequence is a significant sequence for diagnosing osteochondral fractures in HLCFs. MRI can provide important clinical value for treatment decisions of HLCFs without significant displacement.

## Background

Humeral lateral condyle fractures (HLCFs) are common paediatric fractures. It is required to evaluate and diagnose the severity of these fractures timely and accurately in clinical practices, so that patients can avoid severe complications and joint dysfunction (such as nonunion of fractures and delayed union) which have effects on normal growth and development of children’s elbow joints. Frontal and lateral radiographs of the elbow joint are still the first choice when diagnosing children’s HLCFs at present [1]. It is easy to diagnosis children HLCFs with typical morphology and obvious separation and displacement in the end of fracture with traditional elbow joint radiographs, and it is not difficult to identify the fracture line in the HLCFs. However, because the distal humerus epiphysis in children has not yet been fully ossified, radiographs are hard to accurately evaluate and diagnose the damage of articular epiphyseal cartilage, especially in HLCFs without displacement or with micro displacement. It still a big challenge for orthopedic pediatricians and radiologists. For better diagnosis and treatment in HLCFs, some studies tried to use modified radiograph projection, high-frequency ultrasound or arthrography to improve the accuracy of HLCFs' diagnosis, but each examination had its own limitations, and the results were also inconsistent [2–4]. MRI has the advantages of high resolution of soft tissues, multi-directional, multi-parameter imaging, etc. Compared with other imaging methods, MRI has the edge on observing children’s epiphysis and articular cartilage damage. We aimed to investigate: (1) The differences between radiograph and MRI in diagnosing of HLCFs and the integrity of the cartilage chain of the humerus trochlear, and the best MRI sequence to diagnosis HLCFs; (2) The differences in evaluating the degree of fracture displacement in children with HLCFs between radiograph and MRI; (3) The clinical value of MRI in the diagnosis of HLCFs in children without significant displacement.

## Materials and methods

### Patients

The patients in this study were divided into two parts. 60 children with acute elbow joint injuries admitted to the department of orthopedics and pediatrics or emergency department who should be suspected to be HLCFs in clinical practice were included from Dec 2015 to Nov 2017 as the first part patients. Subsequently, 35 HLCFs patients with complete follow-up information who had no obvious displacement (the degree of fractures displacement < 2 mm) on radiograph as the second part patients. The inclusion criteria were as follows: (1) Children aged 0–14 years; (2) Children with acute elbow joint injuries who should be highly suspected or initially diagnosed to be HLCFs in clinical practice; (3) Children with complete medical records and imaging materials; (4) Children with complete follow-up information (part 2 only). The exclusion criteria were as follows: (1) Age > 14 years; (2) Those diagnosed with other types of elbow injuries such as supracondylar fractures of the humerus; (3) Patients with incomplete or missing medical records/imaging materials; (4) Patients who lost to follow-up (part 2 only); (5) Patients were unable to be diagnosed or accurately measured the degree of fracture displacement by MRI due to non-cooperation; (6) Patients with pathological fractures of the elbow joints or combined infections or tumorous lesions.

### Materials and equipment

Digital radiography (GE Discovery XR656) was used to take standard frontal and lateral images of elbow joints (Tube voltage: 60 kV; Tube current: 5mAs; SID: 100 cm; FOV: 24 × 30 cm). GE 3.0 T MR (discovery MR 750; GE; USA) with eight-channel phased array shoulder joint coil was also used to scan elbow joints. MRI scan sequence parameters of elbow joints was showed in Table 1. SYNAPSE system (Dicom version 3.0, FUJI Film Medical System, Stamford, USA) was used to observe the radiograph of the elbow and measure the degree of fractures. GE Workstation (AW46.2, GE, USA) was used to observe cartilaginous fracture and measure the degree of fractures.

**Table 1:** MRI scan sequence parameters of children HLCFs

	Coronal FS-T2WI	Coronal FS-PDWI	Coronal 3D-FSPGR/3D-FS-FSPGR	Sagittal FS-T2WI	Axial FS-T2WI
TR (ms)	3000	1650	7.8/13.6	3000	3000
TE (ms)	108	47	3.3/4.0	102	150
Thi (mm)	2.5	2.5	2.5/2.5	2.5	2.5
Gap (mm)	0.3	0.3	0/0	0.3	0.3
FOV (cm)	14	14	18/18	14	12
NEX	2	2	2/2	2	2
FA (°)	142	142	20/20	142	142
Matrix	320 × 224	320 × 224	320 × 224/320 × 224	320 × 224	320 × 224
Scan Time (s)	66	77	60/62	66	52

HLCFs, Humeral lateral condyle fractures; TR, Time of repetition; TE, Time of echo; Thi, Slice thickness; FOV, Field of view; NEX, Number of excitations; FA, Flip angle

### Methods

All 60 patients who were suspected to be HLCFs accepted routine radiograph examination of the elbow joint in the outpatient or emergency department to diagnose the elbow joint injuries firstly. Then long arm plaster slab external fixation was performed on these HLCFs. Finally, MRI was used to confirm these 60 elbow joint injuries after obtaining parental consents. Taking the fracture line observed during surgery or the appearance of callus repair during the conservative treatment period as the clear fracture criterion, the sensitivity and specificity of radiograph and MRI in the diagnosis of HLCFs and the integrity of the humeral trochlear cartilage chain were calculated respectively, and compared the sensitivity and specificity to confirm whether there were differences between radiograph and MRI. Calculated the sensitivity and specificity of each scan sequence of MRI in the diagnosis of HLCFs osteochondral fractures, and compared whether there were differences. For children diagnosed with HLCFs on both radiograph and MRI, the degree of fracture displacement was measured respectively, and the two examination methods were compared to assess whether there were differences in the degree of fracture displacement. Regarding 35 patients in part 2 (the degree of fractures displacement < 2 mm on radiograph), corresponding treatment measures were taken according to the results of the MRI examination. Compared the ratio of surgical treatment, the ratio of secondary fracture displacement and complications between the stable fracture group and the unstable fracture group on MRI.

### Definition of imaging indicators

Diagnosis of fractures by radiograph: The low-density lucent shadow in humeral lateral condyle metaphysis or separation displacement in epiphysis of capitellum was observed by frontal or lateral radiograph of elbow joints. Diagnosis of fractures by MRI: High or low signal fracture line shadow in humeral lateral condyle metaphysis or distal humeral cartilage by MRI with different scan sequence parameters. Diagnosis of integrity of humeral trochlear cartilage chain by radiograph: when the fracture displacement was less than 2 mm, it was considered as intact humeral trochlear cartilage chain; otherwise, it was considered as broken humeral trochlear cartilage chain. Diagnosis of integrity of humeral trochlear cartilage chain by MRI: when the fracture line involved and perforate the trochlear cartilage of the distal humerus, it was considered as broken humeral trochlear cartilage chain. Undisplaced fractures: no separation displacement of humeral lateral condyle fracture or the degree of separation displacement was less than 2 mm by positive side radiograph of elbow joint. Stable fracture: intact structure of distal humerus trochlea cartilage hinge, the lateral condyle fracture line did not involve the humeral trochlear cartilage chain or the lateral condyle fracture line involved but didn’t perforate the humeral trochlear cartilage chain. Conversely, the lateral condyle fracture line involved and perforated the humeral trochlear cartilage chain, cartilage continuity was broken, it was considered as unstable fracture.

### Measured the degree of HLCFs’ displacement by radiograph and MRI

Radiograph: measured the maximum displacement distance of the lateral fracture space (LFS) on the frontal image of the elbow joints; and measured the maximum displacement distance of the posterior fracture space (PFS) on the lateral image of the elbow joints. MRI: selected the layer with the largest fracture gap to measure; measured the maximum displacement distance of the LFS on coronal FS-T_2_WI image; measured the maximum displacement distance of the PFS on sagittal FS-T_2_WI image.

### Image interpretation

Image of radiograph and MRI interpretation were performed by an orthopedic pediatrician and a senior orthopedic radiologist: (1) diagnosed fracture of the lateral humeral condyle and the integrity of the cartilage chain of the humerus trochlear by radiograph and MRI; (2) analyzed whether the fracture line existed in different MRI sequences; (3) measured HLCFs’ displacement distance. Training them to achieve consistency in image interpretation before study started.

### Statistical analysis

Data analysis was performed with the statistical package SPSS 20.0 (SPSS Inc., Chicago, IL). Consistency in the diagnosis of fractures and the integrity of the cartilage chain of the humeral trochlear between radiograph and MRI was evaluated by Cohen’s Kappa: *κ* < 0.40 poor agreement; 0.40 to 0.75 fair‐to‐good agreement; and > 0.75 excellent chance‐corrected agreement. McNemar’s test was used to evaluate the diagnosis differences between radiograph and MRI. Continuous variables were presented as mean ± standard deviation. *t* test was used to analyze continuous variables. Fisher exact test was used to analyze categorical variables. The consistency between two observers was tested by intraclass correlation coefficients (ICC). Statistical difference was set as *P* value < 0.05 (two-sided).

## Results

### Diagnosed HLCFs by radiograph and MRI

60 children with acute elbow joint injuries admitted to the department of orthopedics and pediatrics or emergency department were suspected to be HLCFs in clinical practice. Finally, 55 children were diagnosed by intraoperative fracture line or follow-up callus repair. Consistency in the diagnosis of HLCFs between radiograph and MRI was fair‐to‐good agreement (*κ* = 0.58, *P* < 0.01). Sensitivity of diagnosing HLCFs by MRI was 100%, which was significantly higher than that by radiograph (89.08%, *P* = 0.03). Consistency in the diagnosis of humeral trochlear cartilage chain integrity between radiograph and MRI was poor agreement (*κ* = 0.12, *P* = 0.18). Sensitivity of diagnosing integrity of trochlear cartilage chain by MRI was 96.30%, which was significantly higher than that by radiograph (62.96%, *P* < 0.01). Detailed data was showed in Table 2.

**Table 2:** The comparison between radiograph and MRI when diagnosing children HLCFs and humeral trochlear cartilage chain integrity

Methods	Case	True positive	True negative	False positive	False negative	Sensitivity (%)	Specificity (%)	*P-*value
*HLCFs*^a^								
Radiograph	60	49	5	0	6	89.09	100.00	0.03
MRI	60	55	5	0	0	100.00	100.00	
*Humeral trochlear cartilage chain integrity*^b^								
Radiograph	60	17	30	3	10	62.96	90.90	< 0.01
MRI	60	26	33	0	1	96.30	100.00	

HLCFs, Humeral lateral condyle fractures

^a^*κ* = 0.58, *P* < 0.01

^b^*κ* = 0.12, *P* = 0.18

### Diagnosed HLCFs by different MRI sequences

**Fig. 1:**
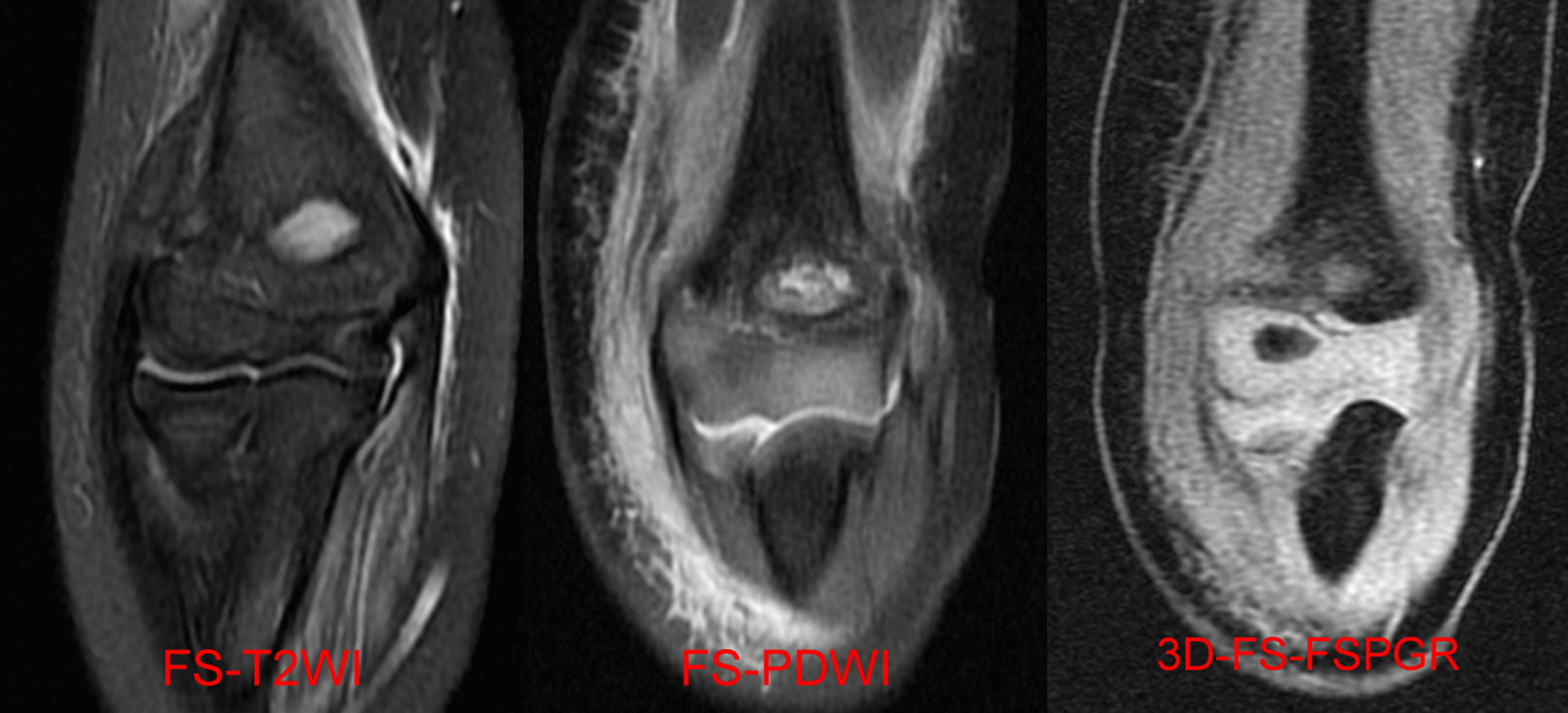
Right elbow coronal sequences in a 5 years old girl with HLCF. FS-T2WI showed the high signal fracture line of the metaphysis of the lateral condyle; FS-PDWI showed that the fracture line was not obvious; neither FS-T2WI nor FS-PDWI showed that the fracture involved the trochlear articular cartilage; the cartilage sensitive sequence 3D-FS-FSPGR showed fracture line partially involved articular cartilage of humeral trochlear. HLCF, Humeral lateral condyle fracture

There were significant differences in sensitivity of diagnosing HLCFs between 3 coronal sequences (*P* = 0.03). After pairwise comparison, it was found that the sensitivity of cartilage sensitive sequence (3D-FS-FSPGR/3D-FSPGR) was different with FS-PDWI and FS-T2WI (*P* = 0.01 and *P* = 0.02, respectively; Fig. 1). But no significant differences were found between coronal, sagittal and axial FS-T2WI. Detailed data was showed in Table 3.

**Table 3:** Comparison of different MRI coronal sequences for the diagnosis of HLCFs in children

Methods	Case	True positive	True negative	False positive	False negative	Sensitivity (%)	Specificity (%)	*P*-value
Coronal 3D-FS-FSPGR/3D-FSPGR	60	55	5	0	0	100.00^a^	100.00	0.03
Coronal FS-PDWI	60	48	5	0	7	87.27^b^	100.00	
Coronal FS-T2WI	60	50	4	1	5	90.91^c^	80.00	

HLCFs, Humeral lateral condyle fractures

Pairwise comparison: ^a^3D-FS-FSPGR/3D-FSPGR VS. FS-PDWI, *P* = 0.01; ^b^FS-PDWI VS. FS-T2WI, *P* = 0.54; ^c^3D-FS-FSPGR/3D-FSPGR VS. FS-T2WI, *P* = 0.02

### Evaluated the degree of HLCFs displacement by radiograph and MRI

49 children were diagnosed as HLCFs by both radiograph and MRI (6 HLCFs who were misdiagnosed by radiograph were excluded). LFS and PFS were measured by radiograph and MRI respectively. Table 4 showed good consistency between 2 observers. The HLCFs’ displacement degree measured by MRI was significantly than that by radiograph (LFS: 2.37 ± 1.38 Vs. 2.20 ± 1.22, *P* < 0.01; PFS: 2.41 ± 1.02 Vs. 2.26 ± 1.03, *P* = 0.01). Detailed data was showed in Table 5.

**Table 4:** Interobserver agreement of measuring LFS (mm) and PFS (mm)

Method	Mean	SD	ICC (*P*)
*LFS*			
Radiograph	2.20	1.22	0.92 (0.01)
MRI	2.37	1.38	0.93 (< 0.01)
*PFS*			
Radiograph	2.26	1.03	0.87 (0.03)
MRI	2.41	1.02	0.90 (0.01)

LFS, Lateral fracture space; PFS, Posterior fracture space

**Table 5:** Paired-samples *t* test between radiograph and MRI when diagnosing children HLCFs

Pairs	Pair’s differences						
	Mean	S D	SE mean	95% CI of the differences		*t*	Sig
				Lower	Upper		
Radiograph LFS–MRI LFS	− 0.17	0.39	0.06	− 0.28	− 0.06	− 3.01	< 0.01
Radiograph PFS–MRI PFS	− 0.15	0.41	0.06	− 0.27	− .031	− 2.54	0.01

HLCFs, Humeral lateral condyle fractures; CI, Confidence interval; LFS, Lateral fracture space; PFS, Posterior fracture space

### The clinical value of MRI in the diagnosis of children HLCFs without significant displacement

**Fig. 2:**
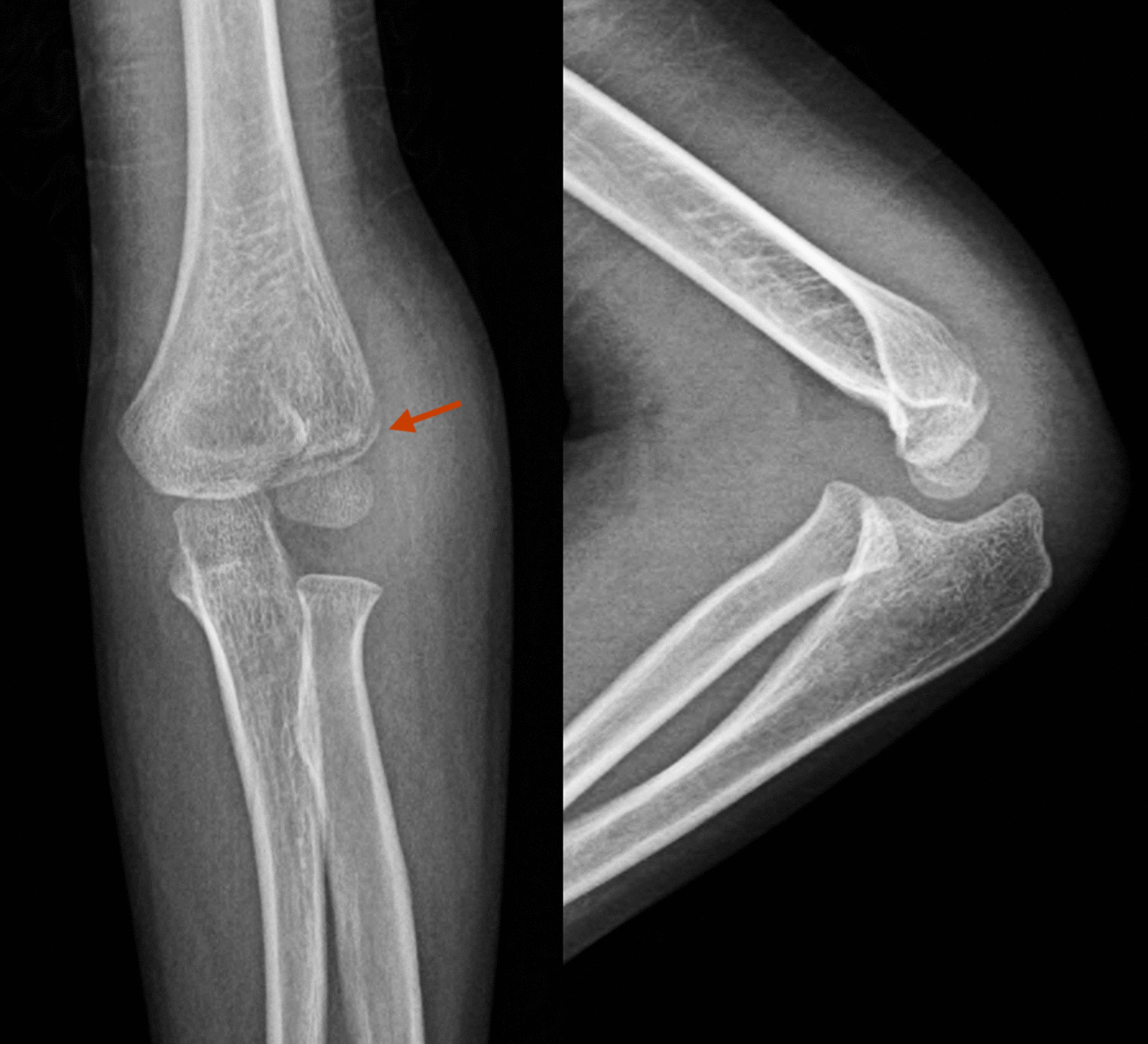
The frontal and lateral radiograph of Left elbow in a 6 years old boy with HLCF. It showed the distance of the lateral fracture space was about 1 mm in frontal image (The arrow showed the fracture line); there was no obvious lateral fracture in lateral image, but positive fat pad sign. HLCF, Humeral lateral condyle fracture

**Fig. 3:**
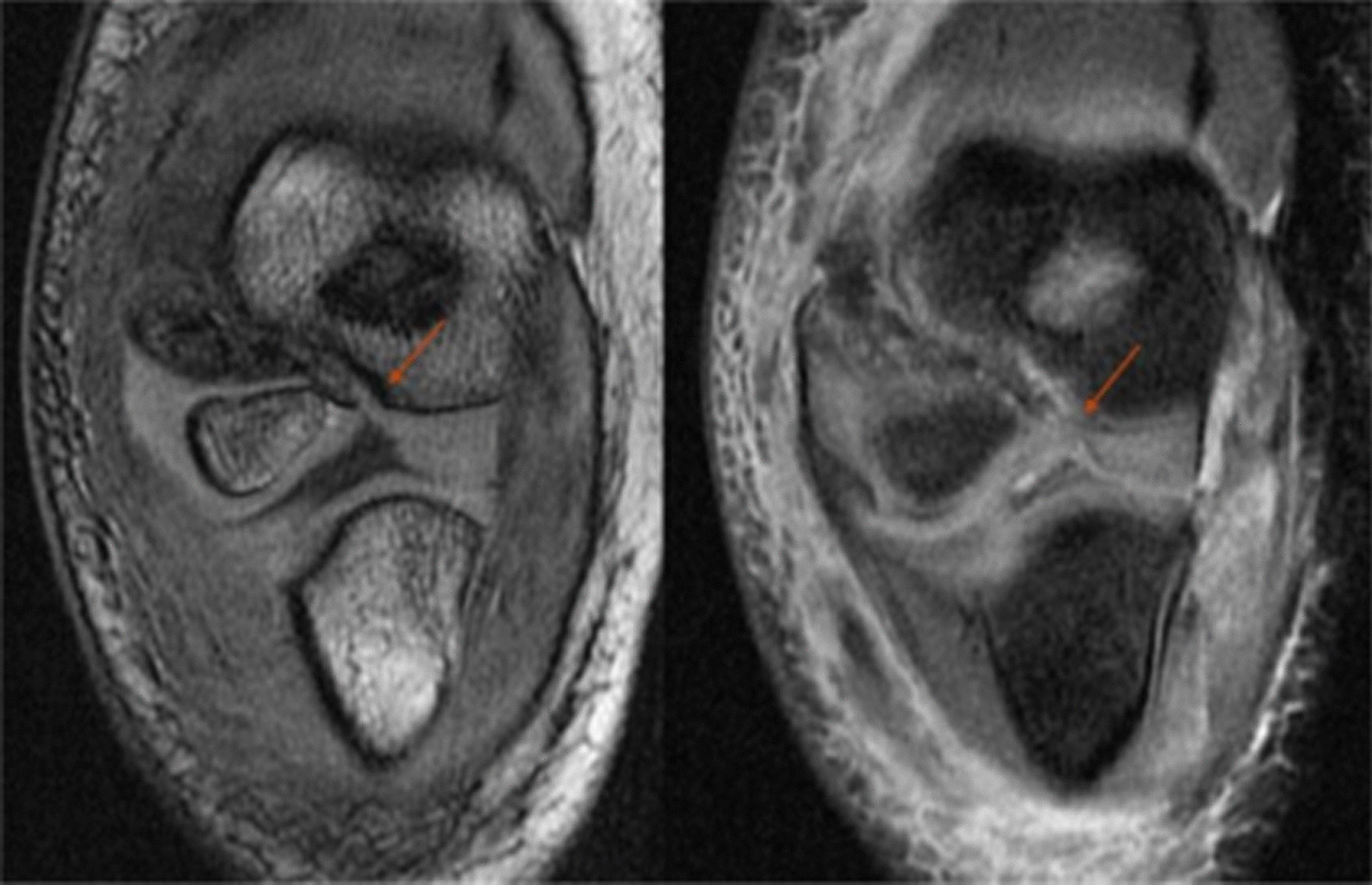
The MRI of right elbow in a 7 years old boy with HLCF. It showed slightly displaced fracture (< 2 mm) by radiograph. MRI showed that the fracture line involved the humeral trochlear cartilage chain, the cartilage articular surface was broken and the fractured end was displaced (3.7 mm) (Left: coronal 3D-FSPGR; Right: coronal T2 fat-suppression; The arrow showed the fracture line). HLCF, Humeral lateral condyle fracture

**Fig. 4:**
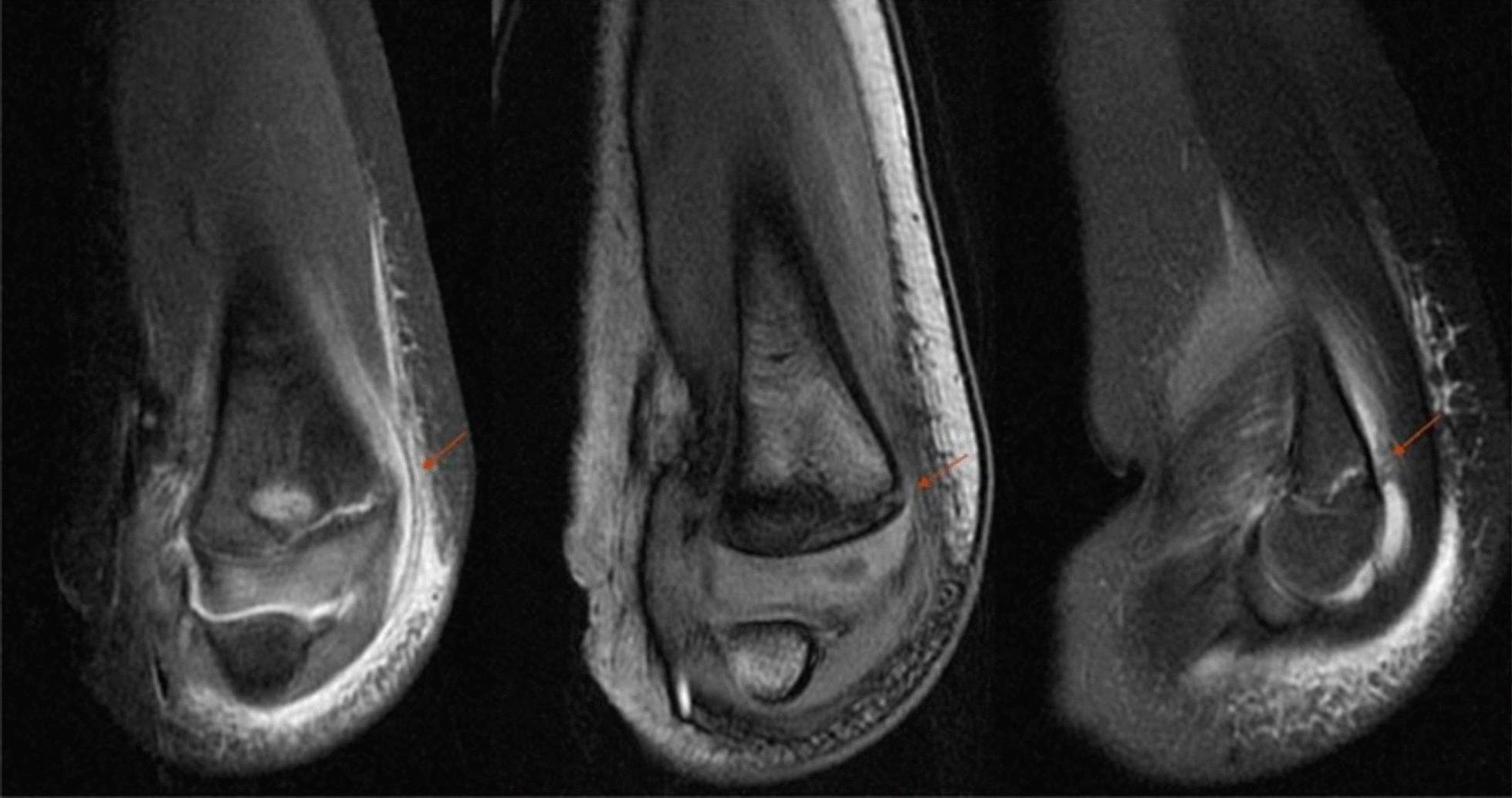
Three years old girl with left elbow HLCF. She was diagnosed undisplaced HLCF by radiograph. MRI showed it was stable fracture and the fracture line was limited to the distal metaphysis of the humerus (Left: coronal FS-T2WI; Mid: coronal 3D-FSPGR; Right: sagittal FS-T_2_WI; The arrow showed the fracture line). HLCF, Humeral lateral condyle fracture

**Fig. 5:**
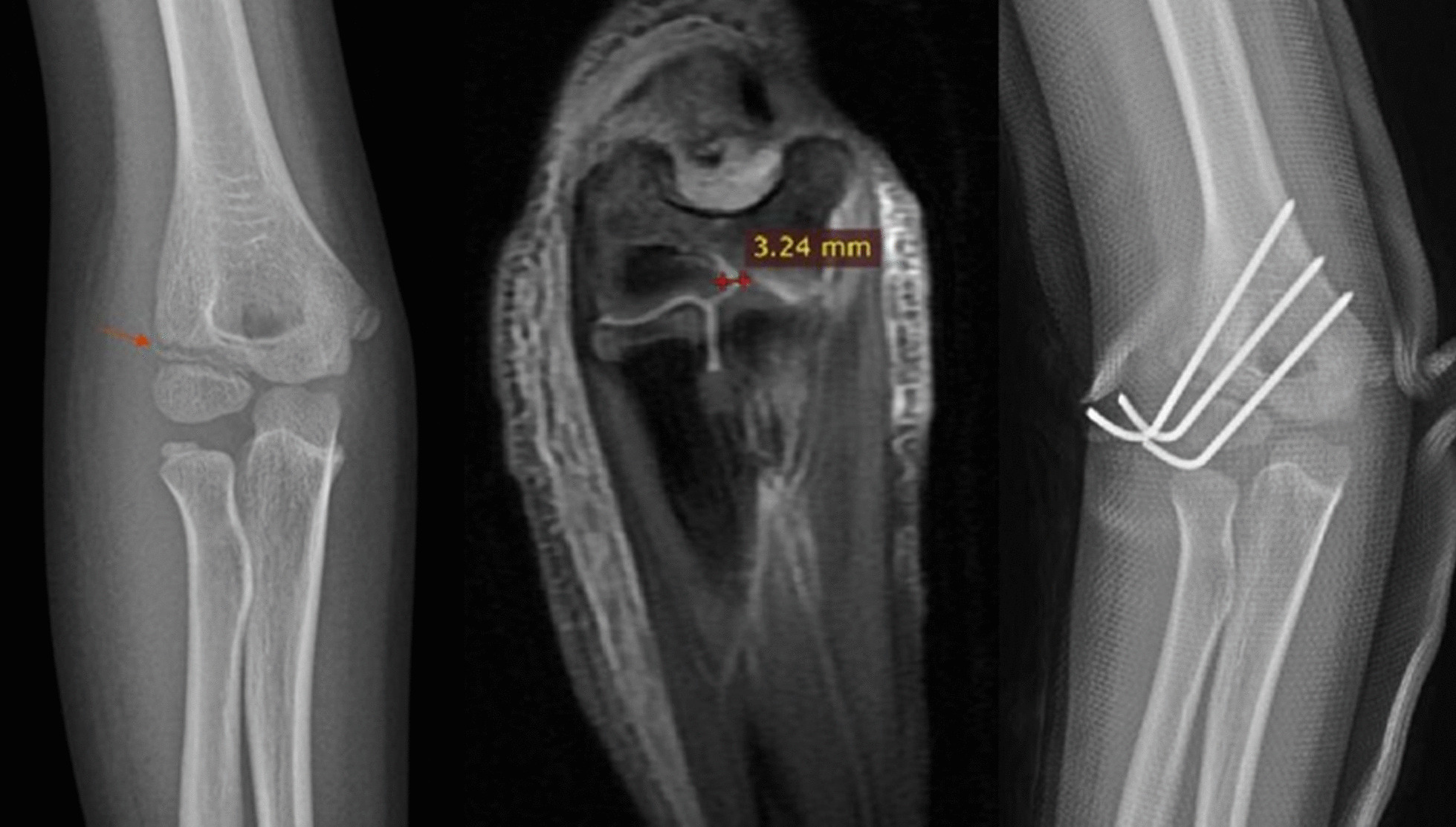
Eight years old boy with right elbow HLCF (Left: It showed slightly displaced fracture (LFS = 1.2 mm) by radiograph; Mid: MRI showed it was unstable fracture and FS-T2WI showed fracture line penetrated the humeral trochlear cartilage chain, and the displacement of articular cartilage was about 3.24 mm; Right: percutaneous K-wire fixation). HLCF, Humeral lateral condyle fracture; LFS, Lateral fracture space; PFS, Posterior fracture space

35 children with HLCFs were included in part 2 study. The average follow-up duration was 10.72 ± 7.64 weeks. These patients were diagnosed as HLCFs without significant displacement (Fig. 2). They were divided into 2 groups according to the image of MRI, 11 patients were unstable fractures (Fig. 3) and 24 patients were stable fractures (Fig. 4). In the unstable fracture group, 5 cases (45.45%) had a fracture displacement of more than 2 mm on MRI (Fig. 5), which was significantly higher than that in stable fracture group (0.00%, *P* < 0.01). Detailed data was showed in Table 6.

**Table 6:** The clinical characteristics between the unstable fracture group and the stable fracture group

	The unstable fracture group (*n* = 11)	The stable fracture group (*n* = 24)	*P*-value
Male (*n*, %)	8 (72.73)	18 (75.00)	0.89
Age (years)	4.71 ± 2.08	5.09 ± 2.93	0.60
Intervals between trauma and radiograph (days)	0.77 ± 0.38	0.83 ± 0.45	0.70
Intervals between trauma and MRI (days)	1.81 ± 0.96	2.04 ± 1.27	0.61
Surgery (*n*, %)	5 (45.45)	0 (0.00)	< 0.01

## Discussion

The incidence rate HLCFs ranks second among elbow fractures in children [5, 6]. As it is the most common intra-articular fracture in pedietric elbow fractures, it is better to achieve anatomical reduction as much as possible to avoid serious complications such as late fracture malunion [7]. Proper operation style is based on timely and correct diagnosis at the early stage of fractures. At present, the preferred examination method is still elbow joint frontal and lateral radiograph, but it does not always accurately diagnose HLCFs, nor can it provide enough information to judge fracture stability to determine the best treatment and prevent secondary fracture displacement [8–10]. Therefore, many other diagnostic methods (such as transverse ultrasonography, arthroscopy, and arthrogram) have been recommended as additional tests to evaluate and diagnose HLCFs in children [11–13]. However, these examinations are invasive and can’t be used routinely for reasons such as the high cost, needing patient sedation, or the poor reproducibility of results. As a non-invasive examination, MRI has the high soft tissue resolution and can identify connective tissue damages such as bone, articular cartilage, ligaments, tendons, and joint capsules. It has been widely used in the diagnosis of osteochondral fractures in acute trauma patients [14, 15]. But there is a lack of studies on the diagnostic value in children HLCFs of MRI.

Currently, the diagnosis, clinical classification and treatment decisions of children with HLCFs still mainly rely on elbow joint radiograph. Various radiograph examination techniques had been proposed in the previous studies to diagnose HLCFs in children, but this also illustrated the difficulty of accurate diagnosis HLCFs and evaluation of fracture displacement by radiograph. The plaster slab will occlude the radiographs and influent its amplification lacks fidelity. When the humeral condyle epiphysis and humeral trochlear epiphysis have not been ossified, the fracture line of HLCFs is often unclear or relatively hidden, which is often confused with distal humerus fracture or elbow joint dislocation. Clarifying the type of fracture and the alignment of the fracture line play a crucial role in determining surgical treatment and the position of Kirschner wire internal fixation. Even if the humeral head is partially ossified, the fracture line can’t be accurately identified by radiograph. Therefore, Manon proposed that neither radiograph nor CT can fully show the position of the cartilage epiphysis, and radiograph can only infer that there may be anatomical abnormalities from the relative displacement of the epiphysis and metaphysis [16].

This study showed that of the 60 children with clinically suspected HLCF fractures, 49 fractures were found by radiograph at the beginning, and 6/11 patients (54.45%) were confirmed to have fractures after follow-up. And MRI images revealed all HLCFs. Gufler et al. [17] performed MRI on 10 children with radiograph negative elbow injuries and found that 5 cases (50.00%) had latent fractures, the proportion was similar to our study. One patient with unstable HLCF was misdiagnosed by radiograph, but he was confirmed by MRI with broken trochlea cartilage chain finally. Then changed the simple plaster external fixation to percutaneous Kirschner wire internal fixation, fracture healing was satisfactory at follow-up. As the incidence of latent fractures in children with HLCFs was high, we proposed MRI should be performed to confirm the diagnosis when patients with elbow swelling after trauma or clinically suspicious HLCFs with negative radiograph.

The integrity of the cartilage chain of the humeral trochlear in children with HLCFs is an important anatomical factor in deciding whether to use surgical treatment or conservative treatment. In our study, 17/49 (34.69%) patients were diagnosed by radiograph as unstable HLCFs with interrupted the integrity of the humeral trochlear cartilage chain, but 26/55 (47.27%) patients were diagnosed by MRI as unstable HLCFs. Sensitivity of diagnosing integrity of trochlear cartilage chain by MRI was 96.30%, which was significantly higher than that by radiograph (63.96%). This may be related to high resolution of soft tissues, and MRI can display osteochondral fractures in multiple layers, multiple directions, and multiple parameters. Our results supported the view that MRI helped to clarify the extent of cartilage fractures and the stability of HLCFs in children [18, 19].

The degree of HLCFs displacement largely determines the treatment. For children HLCFs with a fracture displacement > 2 mm, surgical treatment (such as open reduction internal fixation or closed reduction internal fixation) was performed usually. Long-arm plaster cast external fixation is also effective for HLCFs with no fracture displacement or fracture displacement < 2 mm [3]. However, which treatment to be chosen mainly depends on the accurate assessment of the degree of fracture displacement. Elbow radiographs have always been the first choice for measuring the degree of HLCFs displacement. However, the radiograph of the elbow joint was not sensitive to the measurement of HLCFs displacement in children. Compared with intraoperative observation, the degree of HLCFs displacement by radiograph was often underestimated. Knutsen et al. [20] also confirmed that the real HLCFs displacement distance was greater than the value measured by radiograph through cadaver studies, and the difference between the two ranged from 1.6 to 6.0 mm. Our study showed that no matter whether it was measured by LFS or PFS, there were significant statistical differences between radiograph and MRI. Similar to Knutsen’s [20] cadaver study, the displacement measured by radiograph was less than that by MRI, which was similar to previous study. It is not credible to judge the fracture displacement distance by radiograph alone as the indication for surgery. The degree of fracture displacement measured on MRI images is more accurate, which will facilitate the choice of clinical treatment decisions.

At present, domestic and foreign scholars generally believe that MRI can reflect the pathophysiological changes of articular cartilage through changes in morphology and imaging signals. Therefore, MRI is the best imaging method for non-invasive inspection of articular cartilage [21–23]. However, due to the shortcomings of low signal-to-noise ratio and spatial resolution, conventional MRI sequences such as T1WI or T2WI still have greater limitations for displaying articular cartilage lesions. Cartilage sensitive sequence (3D-FS-FSPGR/3D-FSPGR) was used in our study. The current researches about 3D-FS-FSPGR/3D-FSPGR sequence are mainly focused on the early cartilage degeneration of knee articular, but there are few reports on the application of this sequence to children’s osteochondral fractures, especially for children’s elbow joint cartilage damage. In this study, children’s elbow articular cartilage showed equisignal or slightly higher signal on FS-T2WI and FS-PDWI, while there was obvious high signal on the 3D-FS-FSPGR/3D-FSPGR sequence, and the endochondral fracture lines were low signal in this sequence. Although the 3D-FS-FSPGR/3D-FSPGR sequence is not sensitive to ligament damage and is susceptible to metal artifacts and motion artifacts [24], it is sensitive to articular cartilage fractures. This sequence can provide a reliable basis for the selection of treatment options for children with HLCFs in clinical practice.

Children HLCFs without significant displacement accounts for a large part of the initial diagnosis, and it has been reported there are approximate 33–69% of HLCFs with displacement < 2 mm [25]. Experts have reached a consensus on that open reduction and internal fixation should be performed when HLCFs with fracture displacement > 2 mm. However, for children HLCFs without significant displacement (< 2 mm), there has been controversy over whether to use plaster external fixation or surgical internal fixation. Because children HLCFs may have secondary fracture displacement during conservative treatment. A systematic review reported that 14.9% (53/355) had secondary fracture displacement during conservative treatment [26]. Some scholars even proposed that regardless of whether the fracture was displaced or not, all children with HLCFs required surgical treatment [27]. So far, the diagnosis, clinical classification, and treatment decisions of children with HLCFs have mainly relied on radiographs of the elbow joint. Radiograph assessment of the degree of fracture displacement and location is still the main method for evaluating fracture stability. In the past, it was believed that HLCFs in children with no significant displacement (< 2 mm) on the radiograph were mostly stable fractures, and external fixation treatment could achieve good outcomes. For those with obvious separation and displacement or rotation of the fractures, radiograph can define them as unstable fractures. However, for HLCFs with no obvious displacement, it is difficult to accurately evaluate stability on radiograph alone.

In our study, 31.43% (11/35) HLCFs without significant displacement on radiograph were found to be unstable fractures by MRI. Integrity of humeral trochlea chondral chain was related to fracture stability. We found humeral trochlea chondral chain was completely broken in these 11 patients. Similarly, 28.6% HLCFs were diagnosed with unstable fractures by MRI, although radiograph showed no significant displacement [19]. Before the treatment, the stability of fracture and the degree of fracture displacement were first determined by MRI. MRI found 5 HLCFs patients were unstable fractures with displacement > 2 mm, then changed the original conservative treatment plan which was based on radiograph to surgical internal fixation treatment and achieved bone union. Among the rest 6 patients with external fixation in the unstable fracture group, 2 had secondary fracture displacement, while 24 patients with stable fractures had no secondary fracture displacement during the external fixation treatment. After 35 HLCFs patients were selected for treatment based on MRI, the probability of secondary fracture displacement (5.71%) was lower than that in Vigils’ study (26.87%) [28]. Radiograph was used by the emergency physician in Vigils’ study during the initial emergency department visit, some patients were misdiagnosed, so the probability of secondary fracture displacement was higher. The stability of lateral humeral condyle fractures is related to the integrity of the cartilage hinge and not just on the degree of displacement of the fracture fragment. Radiograph is difficult to accurately evaluate the stability of HLCFs in children without obvious displacement, MRI can clearly display the integrity of the humeral trochlear cartilage chain, and more accurately assess fracture stability and fracture displacement. Therefore, we recommend that children with HLCFs who have no significant fracture displacement perform MRI to provide a reliable reference basis for further clarifying the diagnosis and accurately selecting the treatment plan.

### Limitations

This study mainly focused on children HLCFs without significant fracture displacement. The sample size was relatively small due to various reasons such as the treatment willingness of the children’s guardians, the treatment tendency of the attending physician, or the integrity of the follow-up. Thus, selection bias and follow-up bias were unavoidable. A long-term complication such as fracture nonunion or delayed union were not found in the follow-up. In future studies, follow-up time should be increased to avoid missing the possibility of long-term complications such as cubitus varus, valgus deformity or ulnar nerve palsy. Of course, MRI also has its shortcomings. Although MRI does not have radiation, it avoids the damage of repeated radiograph exposure to children, it is more expensive than radiograph. It will be restricted by the economic conditions of the children’s family. Further study with larger sample size is needed, especially through the comparison of radiograph and MRI to further verify the results of this study.

## Conclusions

In terms of the sensitivity of diagnosing children HLCFs and their stability, MRI is superior to the currently preferred anterolateral and lateral radiograph of elbow joint. Compared with radiograph, MRI can more accurately evaluate the displacement degree of HLCFs in children. The coronal 3D-FS-FSPGR/3D-FSPGR sequence is the most important sequence for diagnosing osteochondral fractures in children HLCFs and its stability. MRI can provide important reference value for clinical treatment decisions of HLCFs without significant displacement, reduce the occurrence of complications, and effectively predict secondary fracture displacement.

## Data Availability

Datasets are available from the corresponding author on reasonable request.

## Abbreviations

HLCFs: Humeral lateral condyle fracture
LFS: Lateral fracture space
PFS: Posterior fracture space
ICC: Intraclass correlation coefficients
